# Analytical performance evaluation of the Mindray enzymatic assay for hemoglobin A_1c_ measurement

**DOI:** 10.1038/s41598-024-63261-y

**Published:** 2024-05-29

**Authors:** Mingyang Li, Xiongjun Wu, Weijie Xie, Yu Zeng, Hui Wang, Han Chen, Anping Xu, Helu Liu, Ling Ji

**Affiliations:** 1 Department of Laboratory Medicine, Peking University Shenzhen Hospital, Shenzhen, Guangdong China; 2 Department of Laboratory Medicine, Shenzhen Integrated Traditional Chinese and Western Medicine Hospital, Shenzhen, China; 3 Department of Clinical Laboratory, Wuhan Asia General Hospital Affiliated to Wuhan University of Science and Technology, Wuhan, China

**Keywords:** HbA_1c_, Enzymatic method, Analytical performance, BS-600M, Diabetes, Laboratory techniques and procedures

## Abstract

Hemoglobin A_1c_ (HbA_1c_) plays a crucial role in diabetes management. We aimed to evaluate the analytical performance of a new enzymatic method kit for HbA_1c_ measurement. The performance of the enzymatic method, including precision, accuracy, and linearity, was evaluated. Moreover, the interference effect from conventional interferents, Hb derivatives, Hb variants, and common drugs were assessed. In addition, the agreement of HbA_1c_ results was compared between enzymatic methods, cation-exchange high-performance liquid chromatography (HPLC), and immunoassays. The intra-assay, between-assay, and total precision of HbA_1c_ were all lower than 2%. HbA_1c_ showed good linearity within the range of 3.96–20.23%. The enzymatic assay yielded results consistent with the external quality control samples, with a bias of less than ± 6% from the target values. The enzymatic method showed no interference from bilirubin, intralipid, vitamin C, Hb derivatives, common Hb variants, as well as antipyretic analgesics and hypoglycemic drugs. The HbA_1c_ results of the enzymatic assay showed good agreement and accuracy compared to those obtained from the HPLC method and the immunoassay. The enzymatic method kit performed on the BS-600M chemistry analyzer is a reliable and robust method for measuring HbA_1c_. It is suitable for routine practice in clinical chemistry laboratories.

## Introduction

Hemoglobin A_1c_ (HbA_1c_) is a modified hemoglobin with a stable adduct of glucose (covalently linked) to the N-terminal valine of the β chain. The formation of HbA_1c_ is a nonenzymatic process that occurs continuously in vivo, starting with glucose in the open-chain form bound to the N-terminus to form aldimines (Schiff bases), which then undergo an Amadori rearrangement to form the more stable ketamine. HbA_1c_ exhibits minimal biological variability and reflects the average blood glucose level over the lifespan of red blood cells^1^. It is strongly associated with the progression of diabetic complications^2,3^. The American Diabetes Association (ADA) and other major organizations have recommended HbA_1c_ for diagnosing diabetes and have adopted a threshold of 6.5% (48 mmol/mol)^4,5^. Therefore, accurate and reliable HbA_1c_ results are important for screening and diagnosing diabetes, monitoring the effectiveness of diabetes treatment, and predicting the risk of diabetes and its complications.

In China, the National Center for Clinical Laboratories (NCCLS) showed that over 70% of laboratories use cation-exchange high-performance liquid chromatography (HPLC) methods, followed by immunoassay, boronate affinity method, capillary electrophoresis (CE), and enzymatic method. These methods make use of the difference in charge between HbA_1c_ and HbA_0_ or the structural differences between glycated and non-glycated forms of hemoglobin^6^. Each method exhibits distinct characteristics and performance and may be affected by factors such as lipemia, anemia, drugs, or hemoglobin variants^7–10^. Therefore, it is necessary to conduct a comprehensive performance evaluation of HbA_1c_ assays prior to their use in the clinical laboratory.

The BS-600M (Mindray, Shenzhen, China) is a newly developed, fully automated biochemistry analyzer that allows for the direct loading of whole blood samples for HbA_1c_ testing using Hemoglobin A1c Kit (Enzymatic Method) at a rate of 120 tests per hour. This study evaluated the analytical performance of a new enzymatic kit conducted on the BS-600M to determine if it meets the requirements of clinical laboratories for HbA_1c_ testing.

## Materials and methods

### Samples

Performance was evaluated using 350 residual whole blood samples in ethylenediaminetetraacetic acid (EDTA)-containing tubes collected from the Laboratory Department of Peking University Shenzhen Hospital. Several small aliquots were made from each fresh sample and stored at − 80 °C prior to analysis. Method comparisons were conducted at Wuhan Asian General Hospital between enzymatic and immunoassay (Roche c501), and at Shenzhen Integrative Medicine Hospital between enzymatic and HPLC (Tosoh G8). The study received approval from the Ethics Committee of Peking University Shenzhen Hospital, and informed consent was waived because there was no information that could help identify individuals. All procedures followed were in accordance with the ethical standards of the responsible committee on human experimentation (institutional and national) and with the Helsinki Declaration of 1975, as revised in 2008.

### HbA_1c_ measurement

HbA_1c_ was measured using the BS-600M automatic biochemistry analyzer (enzymatic assay), the HLC-723 G8 HbA_1c_ analyzer (HPLC method), and the Roche c501 (Immunoassay). All samples were used in the accompanying glycated hemoglobin assay kits.

### The principle of the enzymatic assay kit

The principle of the enzymatic assay kit is as follows: Firstly, red blood cells are lysed in a hypotonic environment. Then sodium nitrite oxidizes Fe^2+^ ions in hemoglobin to Fe^3+^ ions, resulting in the formation of methemoglobin. In the presence of sodium azide, methemoglobin azide is formed, and the absorbance value is measured at 505/800 nm to determine the concentration of total hemoglobin. Protease cleaves the connection between leucine and histidine at the N-terminal end of the β-chain, resulting in the formation of a glycated dipeptide fragment. This glycated dipeptide then undergoes a reaction with fructosyl peptide oxidase, resulting in the production of hydrogen peroxide. Subsequently, under the action of peroxidase, hydrogen peroxide reacts with a colorant, resulting in color shades that are directly proportional to the HbA_1c_ content. The HbA_1c_ content and its percentage in total hemoglobin can be calculated by measuring the absorbance at 660/800 nm.

### Analytical performance

#### Precision

Intra-assay, inter-assay, and total precision were evaluated using high and low-level HbA_1c_ quality control (QC) and fresh whole blood samples. Total precision was evaluated according to the Clinical and Laboratory Standards Institute (CLSI) EP05-A2 guidelines. These samples were mixed and assayed on the BS-600M instrument for 20 consecutive days, with 2 analytical batches per day, 2 tests per analytical batch.

#### Linearity

Linearity was assessed according to the CLSI EP06-A2 guidelines. One sample with a high value (20.23% HbA_1c_) and one sample with a low value (2.96% HbA_1c_) were mixed at different proportions (0%, 6.25%, 12.5%, 18.75%, 25%, 37.5%, 50%, 62.5%, 75%, 87.5%, and 100%) to obtain 11 concentration gradient samples. Each sample was measured on the BS-600M for three times consecutively to obtain measured values and compare them with expected values. The expected HbA_1c_ values were calculated according the following formula: Expected value = (%HbA_1c_ × V_L_ × Hb_L_ + %HbA_1c_ × V_H_ × Hb_H_)/(V_L_ × Hb_L_ + V_H_ × Hb_H_). V_L/H_: volume of the sample with low/high HbA_1c_ value; Hb_L/H_: Hb concentration of the sample with low/high HbA_1c_ value. Hb concentration was measured by a Sysmex XN9000 hematology analyzer (Sysmex Co., Japan).

#### Accuracy

Accuracy was evaluated according to CLSI EP9A2 guidelines by analyzing External quality control (QC) from the International Federation of Clinical Chemistry and laboratory medicine (IFCC, 8 samples) and the National Glycohemoglobin Standardization Program (NGSP, 40 samples). The assay results were compared to the target values and the bias between the two was calculated. The acceptable bias was defined as ± 6.0%. The sigma value for HbA_1c_ was calculated using the formula σ = (TEa − |bias|)/CV.

#### Method comparison

A total of 197 fresh whole blood samples were collected from the Laboratory Department of Shenzhen Integrative Medicine Hospital for the method comparison of BS-600M and Tosoh G8, and 112 fresh whole blood samples were collected from the Laboratory Department of Wuhan Asian General Hospital for the method comparison of BS-600M and Roche c501. The data was analyzed using Passing-Bablok regression analysis and a Bland–Altman plot. Clinical significant bias was defined as a difference exceeding ± 6% at 6% or 9% HbA_1c_^11^.

### Interference

#### Lipemia, Bilirubin, and Vitamin C

The potential error caused by the interfering substance is compared to the allowable error as stated in the CLSI EP7-A2 guideline. Interference was determined by measuring HbA_1c_ in samples containing increasing proportions of lipemia (up to 500 mg/dL), bilirubin (up to 15 mg/dL), and vitamin C (up to 3 mg/dL).

#### Hemoglobin derivatives and hemoglobin variants

Glucose (up to 10,000 mg/dL), aspirin (up to 500 mg/dL), and potassium cyanate (up to 1 mmol/L) were added to whole blood samples. After incubation at 37 °C for 2 h, these samples were assayed to determine the effect of labile A_1c_ (LA_1c_), acetylated hemoglobin, and carbamylated hemoglobin on enzymatic HbA_1c_ determination, respectively. We analyzed samples containing the most common Hb variants (HbS, HbC, HbD, and HbE) to determine the potential bias effect of these variants on HbA_1c_ measurement.

#### Drugs

The effect of medication on the enzyme-based HbA1c assay was analyzed by adding various medications to whole blood specimens. The drug doses were as follows: Paracetamol (0 mg/dL, 50 mg/dL, 100 mg/dL, 150 mg/dL, 200 mg/dL), Ibuprofen (0 mg/dL, 12.5 mg/dL, 25 mg/dL, 37.5 mg/dL, 50 mg/dL), Aspirin (0 mg/dL, 12.5 mg/dL, 25 mg/dL, 37.5 mg/dL, 50 mg/dL), Metformin (0 mg/dL, 1.25 mg/dL, 2.50 mg/dL, 3.75 mg/dL, 5.0 mg/dL), and Acarbose (0 mg/dL, 12.5 mg/dL, 25 mg/dL, 37.5 mg/dL, 50 mg/dL).

#### Hemoglobin concentration

Samples with a hemoglobin concentration range of 30–180 g/L were prepared by mixing erythrocytes and plasma in various proportions. All samples were assayed on the BS-600M and compared to the original HbA_1c_ result.

#### Data analysis

Data were analyzed and plotted using Analyze-it statistical software (version 6.15) and Microsoft Excel 2019. The Kolmogorov–Smirnov test was used to assess the normal distribution of continuous variables. Data that followed a normal distribution were reported as mean and standard deviation (SD). A t-test was used to compare between two groups. Data for variables with skewed distributions were expressed as the median and interquartile range (IQR values). Comparisons between two groups were conducted using the Wilcoxon rank sum test. Categorical variables were expressed as frequencies. A two-sided p-value of lower than 0.05 was considered statistically significant.

## Results

### Analytical performance

#### Precision

When the results of HbA1c were calculated in IFCC units, the coefficients of variation for within-batch precision for the two levels of patient samples were 0.37% and 0.33%, and the coefficients of variation for total precision were 0.67% and 0.40%, respectively, and the coefficients of variation for within-batch precision for the two levels of QC samples were 0.65% and 0.21%, and the coefficients of variation for total precision were 0.87% and 0.35%, respectively. When calculated in NGSP units, the coefficients of variation for within-lot precision for the two levels of patient samples were 0.25% and 0.26%, with total precision coefficients of variation of 0.46% and 0.31%, respectively, and for the two levels of quality-control samples, the coefficients of variation for within-lot precision were 0.38% and 0.17%, with total precision coefficients of variation of 0.51% and 0.27%, respectively (Table 1).

**Table 1:** Intra–assay, inter-assay and total precision of HbA1c measured by enzymatic assay on the BS-600M.

Unit	Samples	Mean Value (%)	N	Intra-assay precision		Inter-assay precision		Total precision	
				SD	CV%	SD	CV%	SD	CV%
NGSP (%)	Sample 1	6.72	80	0.02	0.25	0.02	0.26	0.03	0.46
	Sample 2	9.42	80	0.02	0.26	0.01	0.13	0.03	0.31
	QC 1	5.19	80	0.02	0.38	0.02	0.33	0.03	0.51
	QC 2	10.29	80	0.02	0.17	0.03	0.25	0.03	0.27
IFCC (mmol/mol)	Sample 1	50.00	80	0.19	0.37	0.19	0.38	0.34	0.67
	Sample 2	79.41	80	0.26	0.33	0.13	0.16	0.32	0.40
	QC 1	33.28	80	0.22	0.65	0.19	0.57	0.29	0.87
	QC 2	89.01	80	0.19	0.21	0.28	0.31	0.31	0.35

NGSP, National Glycohemoglobin Standardization Program. IFCC, International Federation of Clinical Chemistry and laboratory medicine. SD, standard deviation. CV, Coefficient of Variation. QC, quality control.

#### Linearity

The relationship between HbA1c and its concentration exhibited excellent linearity across the range of 2.96–20.23%. The regression equation between the theoretical and measured values is Y = 17.15*X + 2.788 (R^2^ = 0.9991), where X represents the theoretical value and Y represents the measured value.

#### Accuracy

A relative bias of ± 6.0% was used as the total allowable error for HbA_1c_^11^. The results of enzymatic assay of whole blood samples of glycosylated hemoglobin assigned by IFCC calibrators and NGSP were correlated with their target values with slopes of 0.9882 and 1.0496 and intercepts of 0.0408 and − 0,3259, respectively, and the relative biases were all within ± 6%. Sigma-metric values for different concentration points were calculated using total precision (CV%) and relative bias at the precision sample mean (Bias%) (Table 2).

**Table 2:** Sigma metrics of HbA1c measured by enzymatic assay on the BS-600M.

Assay	TEa%	Mean value (%, x)	Total precision (CV%)	Accuracy Source	Slope	Intercept	Bias% (y)	Sigma- metric
HbA1c	6.0	6.72	0.46	IFCC standard materials	0.9882	0.0408	− 0.57	11.8
		9.42	0.31				− 0.75	16.9
		5.19	0.51				− 0.40	11.0
		10.29	0.27				− 0.78	19.3
		6.72	0.46	NGSP frozen whole blood	1.0496	− 0.3259	0.11	12.8
		9.42	0.31				1.50	14.5
		5.19	0.51				− 1.31	9.2
		10.29	0.27				1.80	15.6

The total allowable error (TEa) for HbA1c is defined as 6%. The sigma value for HbA1c was calculated using the formula σ = (TEa-|bias|)/CV. Definition of σ: σ ≥ 6 means first-class level; 5 ≤ σ < 6 means "excellent performance"; 4 ≤ σ < 5 means "good performance"; 3 ≤ σ < 4 means "average quality" and σ < 3 means "poor quality".

#### Method comparison

**Figure 1:**
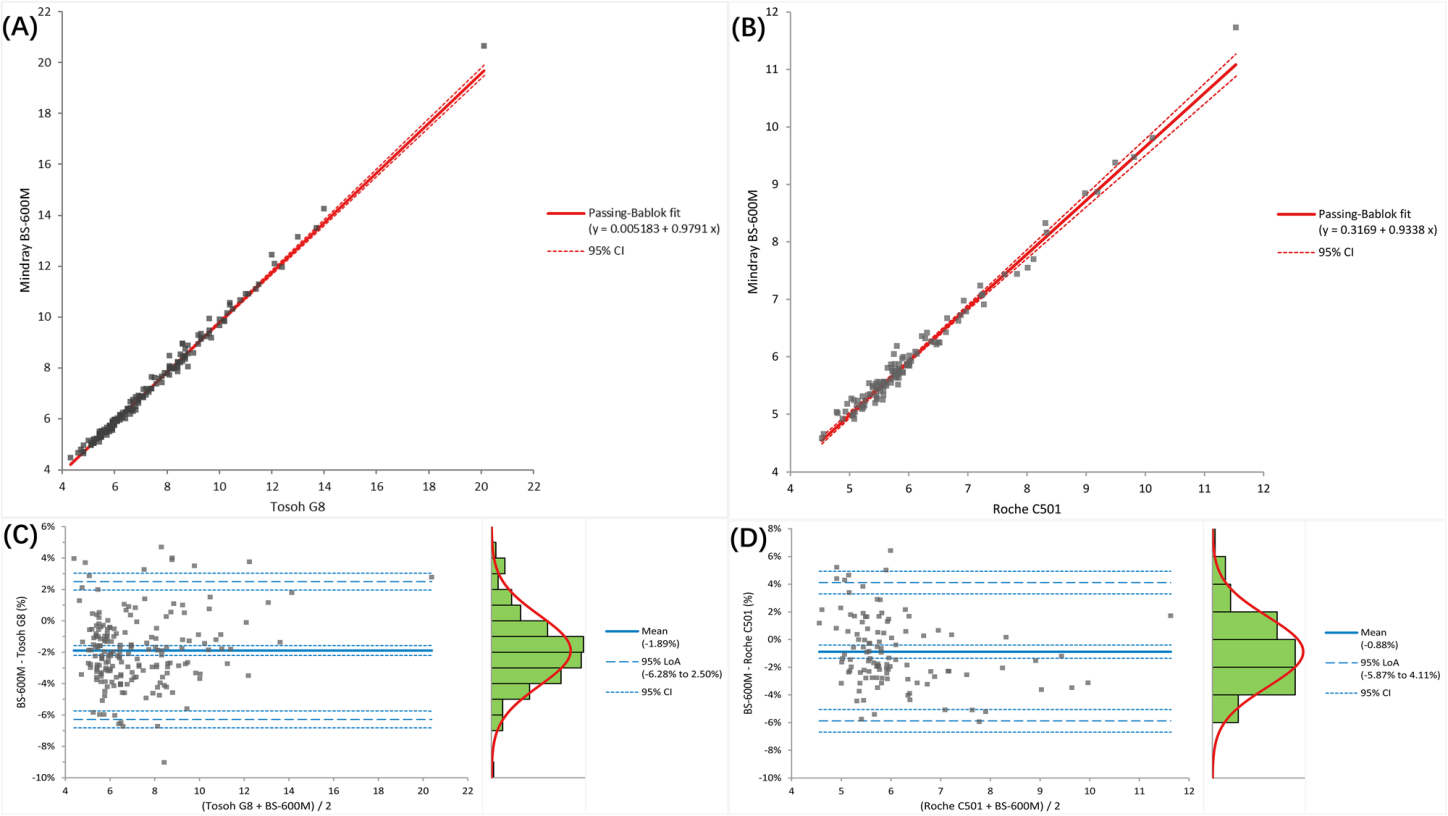
Comparison between BS-600M and other measurement methods. Passing-Bablok regression: (**A**) BS-600M and Tosoh G8; (**B**) BS-600M and Roche c501. Bland–Altman analysis: (**C**) BS-600M and Tosoh G8; (**D**) BS-600M and Roche c501.

The regression equation for BS-600M VS G8 was y = 0.979x + 0.005, with a correlation coefficient of 0.997 (Fig. 1A). The biases at the 6.0% and 9.0% levels were − 2.00% and − 2.03%, respectively (Fig. 1B); The regression equation for the BS-600M VS Roche c501 was y = 0.934x + 0.317, with a correlation coefficient of 0.992 (Fig. 1C). The biases at the 6.0% and 9.0% levels were − 1.34% (− 1.85% to − 0.71%) and − 3.10% (− 4.36% to − 1.91%), respectively (Fig. 1D).

### Interference

#### Lipemia, Bilirubin, and Vitamin C

**Figure 2:**
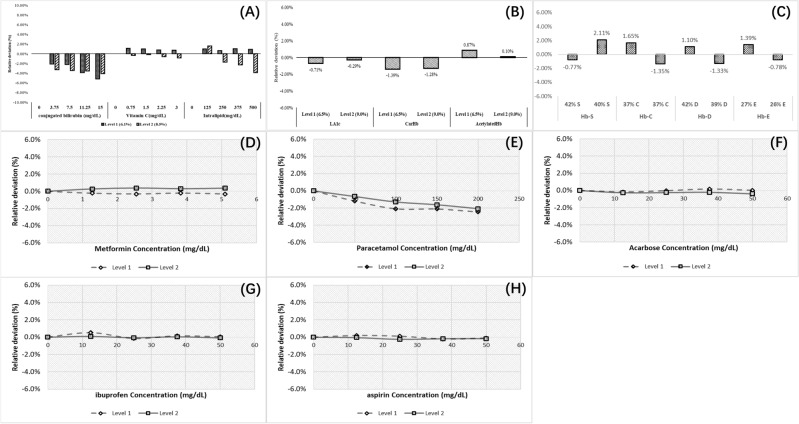
Interferences of HbA1c measured by enzymatic assay on the BS-600M. (**A**) Lipemia, Bilirubin, and Vitamin C; (**B**) LA_1c_, acetylated hemoglobin, and carbamylated hemoglobin; (**C**) Hb S, Hb C, Hb D, and Hb E; (**D**) Metformin. (**E**) Paracetamol. (**F**) Acarbose. (**G**) Ibuprofen. (**H**) Aspirin. Level 1, HbA_1c_ = 6.5%; alevel 2, HbA_1c_ = 9.0%.

The HbA_1c_ (6.1% and 8.5%) biases caused by the Bilirubin (0–15 mg/dL), Vitamin C (0–3 mg/dL), and Intralipid (0–500 mg/dL) were − 3.49% (95% CI − 5.16% to − 2.12%), 0.32% (95% CI − 0.83% to 1.13%), and 0.80% (95% CI − 3.87% to 1.02%), respectively (Fig. 2A).

#### Hemoglobin derivatives and variants

The biases in HbA_1c_ levels (6.5% and 9.0%) caused by LA_1c_, acetylated hemoglobin, and carbamylated hemoglobin were − 0.71% and − 0.29%, − 1.39% and − 1.28%, and 0.87% and 0.10%, respectively (Fig. 2B). The biases caused by HbS, HbC, HbD, and HbE on the enzymatic determination of HbA_1c_ at levels of 5.5% and 7.5% were as follows: − 0.77% and 2.11%, 1.65% and − 1.35%, 1.10% and − 1.33%, 1.39% and − 0.78%, respectively (Fig. 2C).

#### Drugs

The HbA_1c_ results showed a gradual decrease (− 0.66% to − 2.46%) with increasing concentrations of paracetamol, but there was no statistically significant difference (P = 0.1022). Ibuprofen, aspirin, metformin, and acarbose did not have significant effect (P > 0.05) on the enzymatic assay results (Fig. 2D–I).

#### Hemoglobin concentration

**Figure 3:**
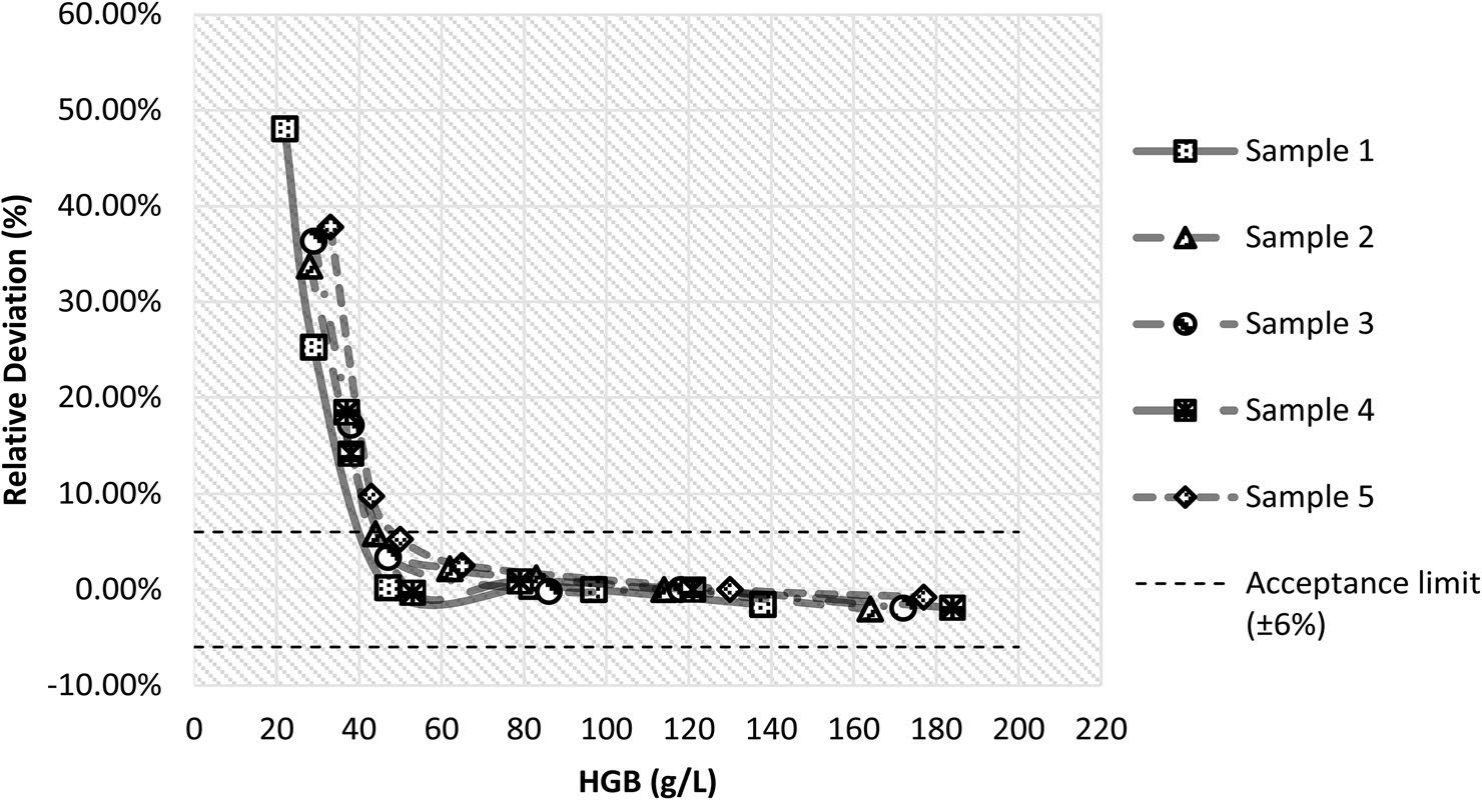
Effects of hemoglobin concentration on HbA1c measured by enzymatic assay on the BS-600M. Dashed lines, ± 6.0% bias. The normal range of hemoglobin concentration is 115–150 g/L for women and 130–175 g/L for men.

When the hemoglobin concentration was ≥ 45 g/L, there was no significant change in the HbA_1c_ results (t = 0.88, P = 0.3890). However, when the hemoglobin concentration was < 45 g/L, the HbA_1c_ levels were significantly higher than the original HbA_1c_ result (t = 4.46, P = 0.0005) (Fig. 3).

## Discussion

With the progress of HbA_1c_ standardization, the discrepancies in HbA_1c_ results from various laboratories and methods around the world are gradually decreasing^11,12^. HbA_1c_ values are used not only for follow-up of glycemic control in diabetic patients but also for diagnosis. Therefore, it is crucial to utilize a testing system that provides accurate HbA_1c_ results for the diagnosis and monitoring of diabetes mellitus. In this study, we evaluated the performance of an enzymatic HbA_1c_ assay. We found that the enzymatic assay performed on the Mindray BS-600M demonstrated good precision, linearity, and accuracy. Moreover, the results obtained with the enzymatic assay were in good agreement with those obtained with the Tosoh HLC-723 G8 and Roche Cobas c501 systems. The performance of the enzymatic method meets the requirements for sample testing in clinical laboratories.

HbA_1c_ is a product formed when hemoglobin and glucose combine in red blood cells. Therefore, any factors that cause changes in the quantity and quality of hemoglobin, such as hemoglobinopathies, derived hemoglobin, abnormalities in the erythrocyte survival lifespan, and drugs, can interfere with the HbA_1c_ assay^13–16^. Our results indicated that routine interferences such as lipemia, hemolysis, jaundice, and vitamin C did not affect the BS-600M enzymatic assay. It is also observed that modified hemoglobins (LA1c, carbamoylated hemoglobins, acetylated hemoglobins) and drugs (paracetamol, ibuprofen, aspirin, metformin, acarbose) did not have a significant impact on the enzymatic HbA1c assay. In addition, the bias of the results of four common hemoglobin variants (Hb S, Hb C, Hb D, and Hb E) did not exceed the permitted range. This demonstrates that enzymatic methods are less susceptible to interference from hemoglobin variants than ion-exchange high-performance liquid chromatography, as previously reported^14^.

The enzymatic assay is based on measuring the percentage of HbA_1c_ in total hemoglobin. Therefore, the concentration of total hemoglobin directly affects the results of the assay. Previous study found that the deviation of sample HbA_1c_ from the target value was less than 0.5% when the hemoglobin concentration was within the range of 9–21 g/dL Total Hb^17^. The results of this study showed that the enzymatic assay of BS-600M provided more stable HbA_1c_ results in the range of HGB ≥ 45 g/L, suggesting that it could be applied to anemia patients (HGB ≥ 45 g/L). It should be noted that the lifespan of red blood cells may change at low Hb concentrations. Comparison of results between assay systems showed good agreement among the enzymatic assay, HPLC, and immunoassay. The expected bias at their medical decision levels was found to be within the acceptable accuracy limit of ± 6%^11^.

In conclusion, the BS-600M enzymatic HbA1c assay demonstrates excellent analytical performance in terms of accuracy, precision, sensitivity and specificity. Compared to HPLC and immunoturbidimetric assay, it shows consistent results and is not adversely affected by common hemoglobin derivatives, drugs and hemoglobin variants. Moreover, this assay provides a rapid detection speed, which makes it a suitable choice for routine practice in clinical chemistry laboratories.

## Data Availability

The datasets used and/or analysed during the current study available from the corresponding author on reasonable request.
